# Characterising the KMP-11 and HSP-70 recombinant antigens' humoral immune response profile in chagasic patients

**DOI:** 10.1186/1471-2334-9-186

**Published:** 2009-11-25

**Authors:** Ivonne D Flechas, Adriana Cuellar, Zulma M Cucunubá, Fernando Rosas, Víctor Velasco, Mario Steindel, María del Carmen Thomas, Manuel Carlos López, John Mario González, Concepción Judith Puerta

**Affiliations:** 1 Laboratorio de Parasitología Molecular, Pontificia Universidad Javeriana, Cra. 7a No. 43-82, Ed. 50, Lab. 113, Bogotá, Colombia; 2 Grupo de Inmunobiología y Biología Celular, Pontificia Universidad Javeriana, Cra. 7a No. 43-82, Ed. 50, Lab. 101, Bogotá, Colombia; 3 Grupo de Parasitología, Instituto Nacional de Salud, Calle 26 No. 51-60, Bogotá, Colombia; 4 Fundación Clínica Abood Shaio, Diag, 110 No. 53-67, Bogotá, Colombia; 5 Departamento de Microbiologia e Parasitologia, Universidade Federal de Santa Catarina, 88040-900, Florianópolis, Santa Catarina, Brazil; 6 Instituto de Parasitología y Biomedicina López Neyra - CSIC - Parque Tecnológico de Ciencias de la Salud. Av del Conocimiento s/n. 18100 Armilla, Granada, Spain; 7 Grupo de Ciencias Básicas Médicas, Facultad de Medicina, Universidad de los Andes, Cra. 1a. No. 18A-10, Bogotá, Colombia

## Abstract

**Background:**

Antigen specificity and IgG subclass could be significant in the natural history of Chagas' disease. The relationship between the different stages of human Chagas' disease and the profiles of total IgG and its subclasses were thus analysed here; they were directed against a crude *T. cruzi *extract and three recombinant antigens: the *T. cruzi *kinetoplastid membrane protein-11 (rKMP-11), an internal fragment of the *T. cruzi *HSP-70 protein_192-433_, and the entire *Trypanosoma rangeli *HSP-70 protein.

**Methods:**

Seventeen Brazilian acute chagasic patients, 50 Colombian chronic chagasic patients (21 indeterminate and 29 cardiopathic patients) and 30 healthy individuals were included. Total IgG and its subtypes directed against the above-mentioned recombinant antigens were determined by ELISA tests.

**Results:**

The *T. cruzi *KMP-11 and *T. rangeli *HSP-70 recombinant proteins were able to distinguish both acute from chronic chagasic patients and infected people from healthy individuals. Specific antibodies to *T. cruzi *crude antigen in acute patients came from IgG3 and IgG4 subclasses whereas IgG1 and IgG3 were the prevalent isotypes in indeterminate and chronic chagasic patients. By contrast, the specific prominent antibodies in all disease stages against *T. cruzi *KMP-11 and *T. rangeli *HSP-70 recombinant antigens were the IgG1 subclass.

**Conclusion:**

*T. cruzi *KMP-11 and the *T. rangeli *HSP-70 recombinant proteins may be explored together in the immunodiagnosis of Chagas' disease.

Polarising the IgG1 subclass of the IgG response to *T. cruzi *KMP-11 and *T. rangeli *HSP-70 recombinant proteins could have important biological effects, taking into account that this is a complement fixing antibody.

## Background

Antibodies against several parasitic antigens are copious during blood-borne parasite infections such as malaria and Chagas' disease. These humoral immune responses have been used for diagnosis, following up individuals throughout the course of a natural infection, vaccination protocols and evaluating drug therapy efficacy. However, little is known about these antibodies' specific role or profile according to disease phases. Concerning humoral responses, it has been described that antibodies against repeat and/or evolutionary conserved sequences are highly predominant in parasitic infections such as that caused by *Trypanosoma cruzi*, the aetiological agent of Chagas' disease [1-3]. B lymphocytes and antigen specific antibodies seem to be crucial for controlling acute infection during the course of *T. cruzi *infection and could determine the fate of the disease's chronic phase [4]. Indeed, B lymphocyte-depleted rats and mice have succumbed to infection with a *T. cruzi *lethal strain [5,6].

Following human infection with *T. cruzi*, some individuals (mostly children) can develop a symptomatic acute phase. However, many individuals recover from acute infection and move on to the indeterminate phase where there are no symptoms and the parasite's persistence can only be indirectly detected by serological tests [7]. This stage of the disease could last several years or even decades. Some indeterminate chagasic individuals progress to chronic disease which unfolds in two major clinical settings compromising either cardiac or digestive tissues [8,9]. Chronic clinical output (cardiopathy or visceral enlargement) seems to be dependent of the host immune response and parasite genetics [8,10,11].

It is becoming apparent that an orchestrated humoral and cellular immune response is needed for controlling *T. cruzi *infection. CD4+ and CD8+ T lymphocytes are essential for eliminating the parasite during the intracellular stage (amastigotes) [10,11] and antibodies acting alone or in association with monocytes operate against extracellular stages (trypomastigotes) [12,13].

With the aim to understand some of the antigen-specific antibody-mediated responses, the profiles of IgG subclass antibody responses and their relationship with the different stages of human Chagas' disease were thus analysed. They were directed against a crude *T. cruzi *antigen and two parasite recombinant proteins: the kinetoplastid membrane protein-11 (KMP-11), a kinetoplastids conserved protein [14,15], and an internal fragment of the *T. cruzi *heat shock protein-70 protein_192-433_(HSP-70T), a highly conserved protein throughout divergent species' evolution [16,17]. The *Trypanosoma rangeli *homologous protein (GenBank accession ABL74477) was also selected to test a full length HSP-70 protein bearing the carboxy terminal GMPG motif which is highly immunogenic in rabbits [18]; it bears 14 copies of the GMPG motif, a higher number compared to the complete *T. cruzi *HSP70 protein (GenBank accession PO5456).

## Methods

### Parasite antigens

Epimastigote lysate was obtained from IRHO/CO/85/MTA Colombian *T. cruzi *I strain in exponential growth in 15% foetal calf serum supplemented-LIT medium (GIBCO, Invitrogen, USA) at 28°C. Parasites were washed twice with cold 1× phosphate-buffered saline (PBS), pH 7.0 and suspended at 1.5 × 10^7 ^parasites/μL in lysis buffer (50 mM Tris -HCl pH 7.0, 1% SDS, 2 mM PMSF, 1% NP40, 5 mM EDTA, 1% β-mercaptoethanol). After sample boiling at 100°C for 10 min and cooling at 4°C for 3 h, the sample was spun for 10 min at 13,000 g and the soluble antigen-containing supernatant was stored at -70°C until use. Protein lysate concentration was determined by Bradford assay and protein profile analysed by 10% sodium dodecyl sulphate-polyacrylamide gel electrophoresis (SDS-PAGE) and Coomassie blue staining [19]. The *T. cruzi *KMP-11 recombinant protein (KMP-11r) [20] and an internal fragment of the *T. cruzi *HSP-70 protein (TcHSP-70T, corresponding to amino acids 192-433), which is conserved and induces functional maturation of murine and human dendritic cells [21,22], were purified as previously described [21]. The entire *T. rangeli *HSP-70 recombinant protein (TrHSP-70) was obtained by cloning in pQE_30 _plasmid (Qiagen, Hilden, Germany) the corresponding encoding region of the Colombian *T. rangeli *Tre strain which was PCR amplified using 70TreATG (5'-CTATAGGATCCATGACGTACGAGGGAGCCA-3') and 70TreTGA specific primers (5'CTATAAAGCTTTCAGTCAACCTCCTCCACCT-3'). Amplification reactions were performed in a final 50 μL volume containing 250 ng purified genomic DNA from the parasite, 1× reaction buffer (10 mM Tris-HCl, pH 9.0, 50 mM KCl, 0.1% Triton X-100), 2 mM MgCl_2_, 0.2 mM of each dNTP, 20 pmol of each primer and 2.6 units of expand high fidelity enzyme (Roche, Mannheim, Germany).

The reaction took place in a MJ Research PTC-100 DNA thermocycler running at 35 cycles having the following amplification profile: 94°C/1 min, 57°C/1 min and 72°C/2:30 min, with final incubation at 72°C for 10 min. Following ligation and transformation, the obtained clone was sequenced and the recombinant protein was over-expressed in *Escherichia coli *after incubation for 2 hours at 37°C with 0.02 mM IPTG. The soluble protein was purified on a Ni^2+^-NTA-agarose affinity column and eluted with phosphate buffer (50 mM NaHPO_4_, 300 mM NaCl) at pH 6.0.

### Patient sera

Four groups of donors were enrolled in the study; all of them participated voluntarily and signed the informed consent form. Seventeen Brazilian acute chagasic (AC) patients from Santa Catarina state who were diagnosed by IgG and IgM immunofluorescence assays were included [23]. Fifty chronic chagasic patients were also enrolled who were positive for anti-*T. cruzi *antibodies in both immunofluorescence assay and ELISA test [24]. They were clinically evaluated at Fundación Abood Clínica Shaio and Instituto Nacional de Salud, Bogotá, Colombia and classified as being 21 indeterminate patients (IND), corresponding to patients with normal findings on electrocardiograms (ECGs) and chest radiographs, and 29 chronic chagasic cardiopathic patients (CCC) who presented abnormal findings on ECGs or/and chest radiographs. Healthy donors (HD) were also included, consisting of 30 people from Colombia who had always lived in non-endemic areas and had negative serology for *T. cruzi*. This study was approved by the Research and Ethics Committees from the Universidad Javeriana's Science Facultad de Ciencias, Pontificia Universidad Javeriana and Fundación Abood Clínica Shaio.

### Detecting anti-*T cruzi *IgG antibodies by ELISA

The ELISA technique was standardised as follows: 96-well immunoassay plates (Nunc Maxisorp, Apogent, USA) were sensitised with 0.1 μg parasite lysate/well or 0.5 μg KMP-11r or 0.1 μg TcHSP-70T and TrHSP-70 recombinant proteins for 3 h at 37°C and subsequently overnight at 4°C. Plates were then washed with 0.05% PBS-Tween (PBS-T). The wells were incubated for 1 h at 37°C with blocking solution (5% nonfat dried milk powder in PBS-T) to avoid unspecific binding. After washing the wells three times with PBS-T sera at 1:100 dilution for lysate, KMP-11r and TrHSP-70 recombinant antigens and 1:50 dilution for TcHSP-70T protein, blocking solution was added to the wells and they were incubated for 2 h at 37°C. Wells were then washed four times with PBS-T and anti-human IgG (γ-chain specific) alkaline phosphatase conjugate (Sigma Chemical Co., St. Louis, MO) was added to blocking solution at 1:15,000 dilution for lysate and KMP-11r and 1:10,000 for TcHSP-70T and TrHSP-70 proteins. Plates were incubated for 1 h at 37°C. After four washes with PBS-T, p-nitrophenylphosphate substrate solution (Sigma Chemical Co., St. Louis, MO) diluted in diethanolamine buffer (pH 9.8) was added to each well. The reaction was developed in the dark at room temperature for 30 min and stopped by adding 0.5 N NaOH. Optical densities (OD) were determined on a Labsystems Multiskan plate reader (Vantaa, Finland) at 405 nm wavelength. Cut-off values were assessed as being mean optical density value in samples from non-chagasic donors plus 3 standard deviations.

### Detecting anti-T cruzi IgG isotypes by ELISA

A 0.1 μg antigen/well concentration for lysate and 0.5 μg/well for KMP-11r and TrTHSP-70 was used in the ELISA technique for IgG1, IgG2, IgG3 and IgG4 subclass antibody detection (except for KMP-11r IgG3 detection where 0.1 μg/well was used).

Samples were diluted at 1:50 for all assays and anti-human IgG1, IgG2, IgG3 and IgG4 peroxidase conjugate (MP Biomedicals, Inc. Ohio, USA) were diluted at 1:500. Substrate solution was *O*-phenylendiamine dihydrochloride (OPD) (Sigma Chemical Co., St. Louis, MO, USA). Readings were taken at 492 nm wavelength.

### Statistical analysis

Differences among groups were assessed by Student's *t *test when n ≥ 10 and by Mann-Whitney test when n < 10. GraphPad Prism 5.0 statistical software was used for both analyses. They were considered to be statistically significant when p < 0.05.

## Results

### Analysing recombinant proteins and parasite soluble proteins

**Figure 1:**
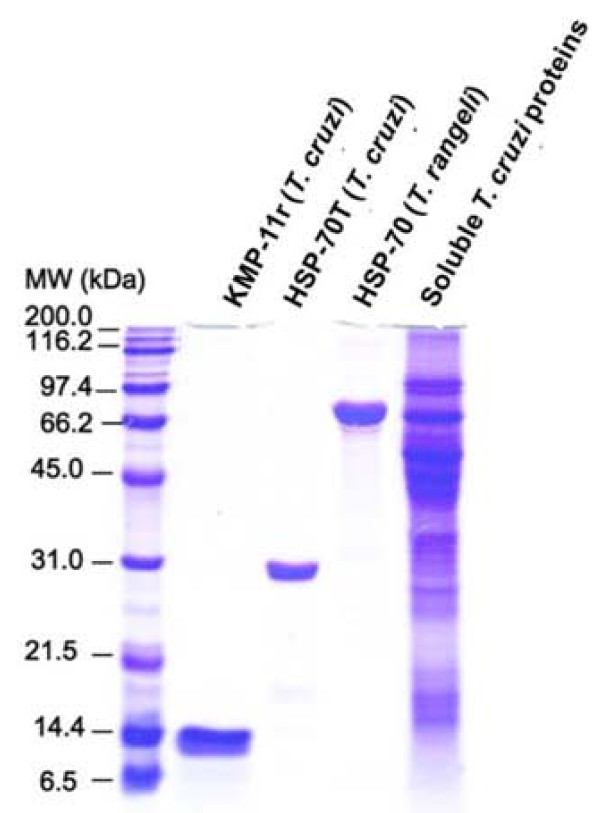
**Purifying recombinant proteins by Ni2+ affinity chromatography and extracting total soluble proteins from *Trypanosoma cruzi***. Purified *T. cruzi *KMP-11 recombinant protein (KMP-11r), *Trypanosoma cruzi *truncated HSP-70 protein (TcHSP-70T), *Trypanosoma rangeli *HSP-70 recombinant protein (TrHSP-70), and crude *T. cruzi *lysate were electrophoresed in 12% SDS-PAGE and stained with Coomassie blue. Lane MW corresponds to molecular weight markers shown in kDa.

The KMP-11r, TcHSP-70T and TrHSP-70 recombinant proteins as well as *T. cruzi *total crude antigen from the parasite were analysed by SDS-PAGE (Figure 1). As expected, bands of around 14, 30, and 76 kDa were observed for KMP-11r, TcHSP-70, and TrHSP-70 recombinant antigens, respectively. Purity was >95% as assessed by Coomassie blue staining.

### Detecting anti-*T. cruzi *IgG antibodies

**Figure 2:**
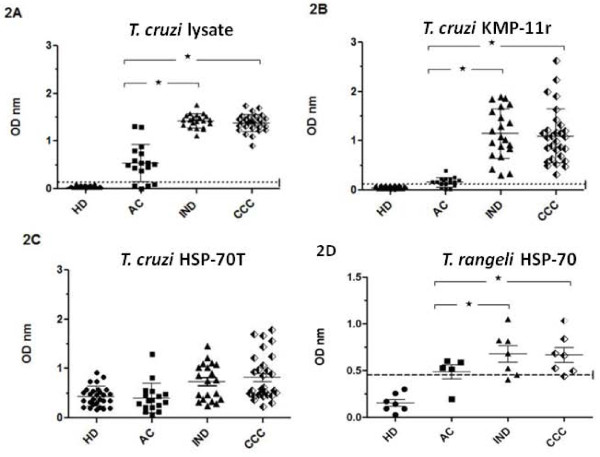
**ELISA for total IgG antibody levels from acute (AC), indeterminate (IND) and cardiac chronic (CCC) chagasic patients and healthy individuals (HD) against *T. cruzi *lysate (2A), KMP-11 (2B), truncated *T. cruzi *HSP-70 (TcHSP-70T) (2C), and *T. rangeli *HSP-70 (2D) recombinant proteins**. Values are given as optical densities at 405 nm. The dotted line represents the cut-off values based on the mean of healthy individual values plus 3 standard deviations. Horizontal lines on each group represent mean and standard deviation values, the mean being the larger one. Statistically significant differences among chagasic groups are represented by an asterisk.

The results revealed that 76.5% (13 out of 17) of the patients in the acute phase and 100% of those in the indeterminate (No = 21) and cardiopathic (No = 29) chronic stages recognised *T. cruzi *lysate proteins. Healthy donors did not display reactivity against crude parasite antigen. There was a statistically significant higher mean OD for anti-*T. cruzi *IgG levels in sera from patients in both chronic stages (IND = 1.424 ± 0.14, and CCC = 1.384 ± 0.18) when compared to that from the acute group (0.539 ± 0.38), p < 0.0001, Figure 2A).

Figure 2B shows the reactivity of sera from all patient groups against the KMP-11 recombinant protein. Healthy donors did not recognise the KMP-11r protein. The percentage of chagasic patients presenting specific anti-KMP-11r protein IgG antibodies having a reactivity value above cut-off level was 64.7% (11 out of 17) in the acute group and 100% for both chronic indeterminate and cardiopathic groups. The mean OD value of IgG reactivity against the KMP-11r protein was higher in indeterminate patients (1.148 ± 0.508) than that found in the acute group (0.163 ± 0.096, p < 0.0001). Likewise, chronic chagasic cardiopathic patients also had a higher reactivity level (1.1 ± 0.55) than that observed in the acute group (p < 0.0001); 35.3% (6 out of 17) acute, 66.6% (14 out of 21) indeterminate and 86.2% (25 out of 29) chronic chagasic cardiopathic patients presented anti-TcHSP-70T reactivity values higher than the mean value for reactivity sera. However, it is worth mentioning that healthy donors recognised the *T. cruzi *HSP-70T recombinant protein, showing significant reactivity with absorbance values ranging from 0.161 to 0.922 (0.438 ± 0.201 mean) (Figure 2C).

The TrHSP-70 recombinant protein was assayed to test the recognition of full length HSP-70 by sera from healthy donors and chagasic patients at different stages of the sickness. Sera from 5 Brazilian acute and 14 Colombian chronic patients (7 indeterminate and 7 cardiopathic) were thus also tested for the presence of anti-TrHSP-70 protein antibodies (Figure 2D). The results showed that most patients in acute (4 out of 5), indeterminate (5 out of 7) and chronic (6 out 7) phases had specific anti-*T. rangeli *protein antibodies. Remarkably, sera from healthy donors did not recognise the *T. rangeli *HSP-70 protein at assayed sera dilution.

**Figure 3:**
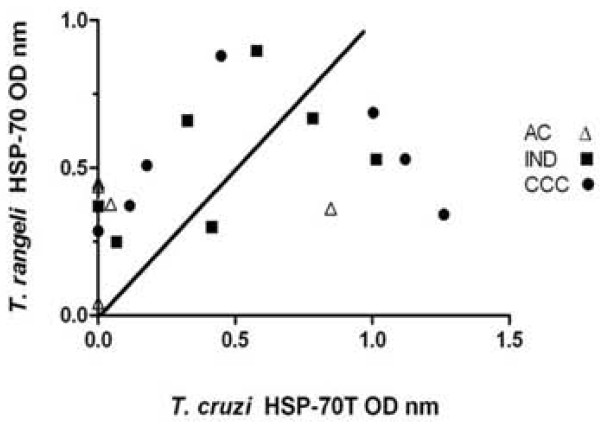
**Comparing normalised optical densities (OD) from from sera evaluated for TcHSP-70T and TrHSP-70 recombinant proteins**. The ELISA for the TcHSP-70T recombinant protein was performed with sera diluted 1/50 and 1/100 for the TrHSP-70 antigen. Data was normalised by subtracting the mean OD of the healthy group for each protein and, when mean OD obtained was lower than 0, it was fixed as 0.

Their absorbance values were compared to analyse the relationship of sera reactivity against both *T. cruzi *and *T. rangeli *HSP-70 recombinant antigens. Absorbance values were thus normalised by subtracting healthy donors' mean OD values and comparing them on a graph. Figure 3 shows that 11 out of 19 chagasic patients had higher reactivity against TrHSP-70 than TcHSP-70. On the other hand, only 7 patients (1 out of 5 acute, 3 out of 7 indeterminate and 3 out of 7 cardiopathic groups) exhibited higher reactivity against TcHSP-70 than TrHSP-70.

### Anti-*T. cruzi *antibody IgG subclass profile

**Figure 4:**
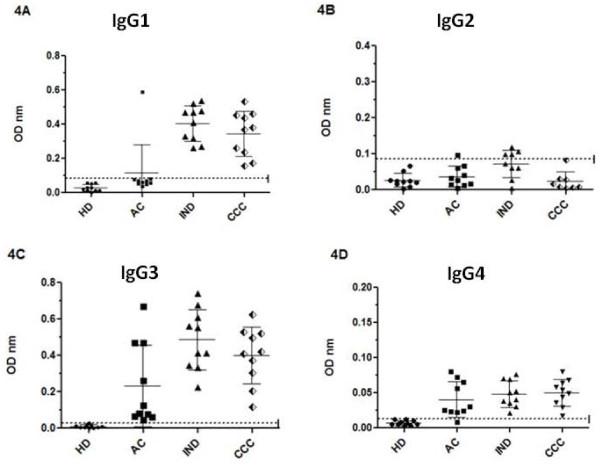
**IgG isotype profile against *T. cruzi *lysate**. IgG1 (4A), IgG2 (4B), IgG3 (4C) and IgG4 (4D) isotype levels for patients in acute (AC), indeterminate (IND) and cardiac chronic phase (CCC) and healthy individuals (HD). Values are given as optical densities at 492 nm. The dotted line represents the cut-off values based on the mean of healthy individual values plus 3 standard deviations. Horizontal lines on each group represent mean and standard deviation values, the mean being the larger one.

**Figure 5:**
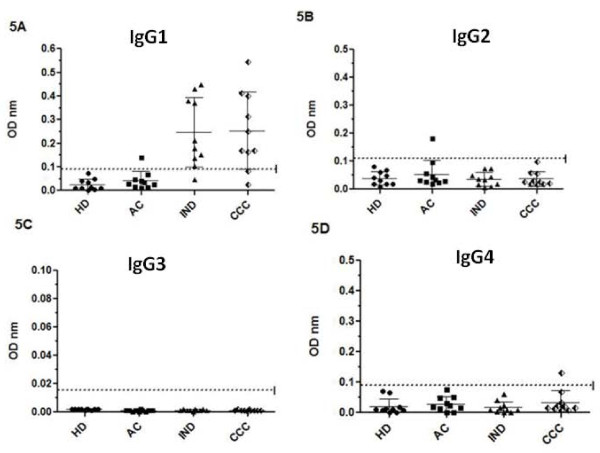
**IgG isotypes profile against *T. cruzi *KMP-11 recombinant protein**. IgG1 (5A), IgG2 (5B), IgG3 (5C) and IgG4 (5D) isotype levels for patients in acute (AC), indeterminate (IND) and cardiac chronic phase (CCC) and healthy individuals (HD). Values are given as optical densities at 492 nm. The dotted line represents the cut-off values based on the mean of healthy individual values plus 3 standard deviations. Horizontal lines on each group represent the mean and standard deviation values, the mean being the larger one.

**Figure 6:**
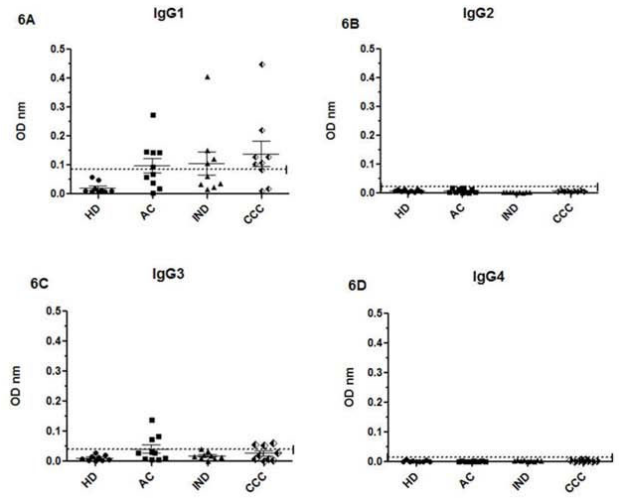
**IgG isotypes profile against *T. rangeli *HSP-70 recombinant protein**. IgG1 (6A), IgG2 (6B), IgG3 (6C) and IgG4 (6D) isotype levels of patients in acute (AC), indeterminate (IND) and cardiac chronic phase (CCC) and healthy individuals (HD). Values are given as optical densities at 492 nm. The dotted line represents the cut-off values based on the mean of healthy individual values plus 3 standard deviations. Horizontal lines on each group represent the mean and standard deviation values, the mean being the larger one.

KMP-11 and TrHSP-70 recombinant proteins were measured in sera from ten patients who exhibited IgG levels higher than the cut-off value for all antigens used to help analyse IgG1, IgG2, IgG3 and IgG4 subclass levels against *T. cruzi *lysate (Figures 4, 5, 6).

When the IgG antibody subtype was tested against parasite soluble proteins it was observed that IgG1 and IgG3 were the predominant subclasses in chagasic patients (Figure 4). Indeed, all sera from chagasic patients (acute, indeterminate and cardiopathic) presented IgG3 isotype antibodies against crude parasite lysate. Interestingly, IgG1 was detected in both groups of chronic chagasic patients (indeterminate and cardiopathic) having similar mean OD values (IND: 0.405 ± 0.1, CCC: 0.345 ± 0.13). These values were higher than those detected from patients in the acute phase (p = 0.0002 and p = 0.0016, respectively). A low IgG4 response was observed in all chagasic patients (0.08 maximum OD). Most chagasic patients presented an anti-lysate IgG2 reactivity OD below the cut-off value.

Figure 5 shows the IgG subclass profile for KMP-11 recombinant antigen. The results indicated that the antibodies generated against KMP-11r by chagasic patients were of the IgG1 subtype. There were no detectable anti-KMP-11 specific IgG2, IgG3 or IgG4 subclass antibodies with the exception of one serum from a patient in acute phase (subtype IgG2) and another in a chronic cardiopathic patient (subtype IgG4); the antibody titre proved low in both cases.

When the IgG subtype of anti-*T. rangeli *HSP-70 recombinant protein antibodies was studied it was observed that HSP-70 specific antibodies were predominantly of the IgG1 isotype in chronic chagasic patients (both indeterminate and cardiopathic) (Figure 6). IgG3 subclass antibodies were detected in some acute and chronic cardiopathic patients.

## Discussion

Chagas' disease, before becoming confined to Latin-America, had reached the North-American and European continents through immigration, thus becoming a health problem in those non-endemic countries [25]. This is an intriguing parasitic disease due to its chronic natural evolution. However, only 30% of infected individuals during the indeterminate stage develop later heart or digestive tissue damage, suggesting an early infection control which may be related to the immune system and also to the parasite's strain and the host's genetic factors [8,26]. A specific antibody response seems to play an important role in controlling early parasite blood stages as demonstrated by passive transfer of immune serum [27], monoclonal antibodies [28] or B cells to depleted rats [5]. Dissecting protective antibody targets against parasite infection has proved difficult because of the large number of parasite genes (12,000 genes per haploid genome), from which at least 1,500 have been cloned [3].

The antibody profile of total IgG and IgG subclasses against parasite lysate and KMP-11 and HSP-70 recombinant proteins was evaluated to understand the course of antibody response during *T. cruzi *infection in acute, indeterminate and cardiopathic chronic chagasic patients. The results showed that the number of individuals recognising crude parasite antigen was significantly higher in patients in indeterminate and chronic phases than those in the acute phase. Moreover, indeterminate and chronic patient sera titre was also higher compared to that of acute patients. The consistently higher IgG response in chronic patients occurring with all the antigens used was also observed. The difference was even more notable with the response to the KMP-11 recombinant protein in which a very low response was found during the acute phase. K1 (an amino-terminal peptide from this protein) also induced significant reactivity in both groups of Colombian chronic chagasic patients [29]. The data observed here are consistent with that previously described in humans and animal models where high levels of total IgG specific response has been a hallmark of the sickness' chronic stages [30,31].

The acute sera tested in the present study were from patients involved in an outbreak of acute Chagas' disease due to ingesting sugar cane juice where *T. cruzi *TcII lineage was identified in nine patients [23]. It is possible that the low reactivity observed in the acute phase group might have been due to the early stage of infection (60 days) since all patients presented high anti-*T. cruzi *IgM antibody titres (1:320) in immunofluorescence test. Another explanation might be supported by the lineage of the *T. cruzi *strain implicated. It has been recently described that mice infection with *T. cruzi *TcI lineage, the main circulating lineage in Colombia [32], is correlated with higher IgG responses compared with the *T. cruzi *TcII ones [33].

The highly conserved chaperone HSP-70 is a major cloned sequence of pathogens which can induce cross-reactive responses [34]. A fast acute chagasic patient IgG response to TcHSP-70 may be explained by pre-existing specific memory T cells, elicited by other HSP-70 pathogens. Another non-exclusive explanation refers to the mitogenic capability of HSP-70 for B cells which has been reported for *Leishmania infantum *HSP-70 proteins in rodent models [35]. Similar to that reported by Requena *et al*., (1993) using the whole HSP-70 parasite protein, sera from healthy individuals in this study showed significant reactivity against the *T. cruzi *truncated protein (TcHSP-70T). This result was not surprising taking into account that the truncated version's identity with the same region in the human HSP-70 protein was a little bit higher (74%) than when comparing the whole protein (70%). Moreover, the truncated version lacks the C- terminal region in which some parasite specific epitopes are located [34].

It is particularly worth noting that the response against the complete *T. rangeli *HSP-70 protein was characterised by higher reactivity in most chagasic patient sera, along with the lack of it in healthy individuals.

This finding may have resulted from several GMPG motifs being present at the *T. rangeli *HSP-70 protein's C-terminal region. Besides, it has been demonstrated recently that some *T. rangeli *proteins can elicit a strong humoral response in chagasic patients [36]. Collectively, these results suggest that *T. rangeli *could be one of the pathogens which can boost antibody response to this antigen, the HSP-70 protein seems to be involved in cross-reaction between these trypanosomes and that the *T. rangeli *HSP-70 protein could be explored for diagnosis strategies.

IgG subclasses were then measured against total crude lysate and two recombinant proteins in all sera groups to determine the relationship with disease stage. The data showed that the specific antibodies in the acute patients were from subclasses IgG3 and IgG4 against crude *T. cruzi *antigen whereas IgG1 and IgG3 were the prevalent isotypes in indeterminate and chronic chagasic patients. These findings agreed with reported high prevalence for IgG3 and IgG1 isotypes against epimastigotes in all clinically severe stages of Argentinean and Brazilian chronic chagasic individuals [37,38]. By contrast, Mexican chagasic donors suffering cardiopathy had more IgG1 and IgG2 and less IgG3 [39]. Moreover, anti-*T. cruzi *IgG2 association with cardiac involvement or chronic cardiac disease severity has been reported in the Brazilian and Mexican chagasic population, respectively [38,39]. These apparently disagreeing results may have been due to differences in the circulating parasite strain in each area. Indeed, it has been shown recently that parasite genotype can affect IgG subclass profile [33].

The specific prominent antibodies in all disease stages against *T. cruzi *KMP-11 and *T. rangeli *HSP-70 recombinant antigens were only from the IgG1 subclass. These results were similar to those reported by Trujillo *et al*., (1999) using the *Leishmania panamensis *KMP-11 protein [40].

Likewise, when an epimastigote acidic antigenic fraction was used as antigen, IgG1 was the main antibody isotype detected by ELISA in all Argentinean chronic patients [37]. On the contrary, the distribution of IgG subclasses against epimastigote ribonucleoproteins and the cytoplasmic (CRA) and flagellar (FRA) recombinant repetitive antigens in chronic chagasic patients assessed by ELISA were shared by IgG1 and IgG3 [41,42]. Since, IgG1 is a complement fixing antibody which also mediates cooperative function with phagocytes through their Fc receptors [43], then this specific antibody's role in controlling the infection or the disease progressing in severity needs to be addressed.

## Conclusion

The *T. cruzi *KMP-11 and the *T. rangeli *HSP-70 recombinant proteins may be explored together in Chagas' disease immunodiagnosis. Polarising the IgG response IgG1 subclass to *T. cruzi *KMP-11 and *T. rangeli *HSP-70 recombinant proteins could have important biological effects, taking into account that this is a complement fixing antibody.

## Competing interests

The authors declare that they have no competing interests.

## Authors' contributions

CP, JG, MCT, MCL and AC conceived and designed the study. ZC, FR and VV clinically evaluated the patients and controls. IDF performed the experiments. CP, JG, AC, MCT, MCL and MS interpreted the results and wrote the manuscript. All authors have read and approved the final manuscript.

## Pre-publication history

The pre-publication history for this paper can be accessed here:

http://www.biomedcentral.com/1471-2334/9/186/prepub
